# Targeted drug delivery using nanobodies to deliver effective molecules to breast cancer cells: the most attractive application of nanobodies

**DOI:** 10.1186/s12935-024-03259-8

**Published:** 2024-02-10

**Authors:** Mohadeseh Haji Abdolvahab, Pegah Karimi, Nasrin Mohajeri, Mohammad Abedini, Hamed Zare

**Affiliations:** Recombinant Proteins Department, Breast Cancer Research Center, Motamed Cancer Institute, ACECR, Tehran, Iran

**Keywords:** VHH, Nanobody, Drug delivery, Breast cancer

## Abstract

Targeted drug delivery is one of the attractive ways in which cancer treatment can significantly reduce side effects. In the last two decades, the use of antibodies as a tool for accurate detection of cancer has been noted. On the other hand, the binding of drugs and carriers containing drugs to the specific antibodies of cancer cells can specifically target only these cells. However, the use of whole antibodies brings challenges, including their large size, the complexity of conjugation, the high cost of production, and the creation of immunogenic reactions in the body. The use of nanobodies, or VHHs, which are a small part of camel heavy chain antibodies, is very popular due to their small size, high craftsmanship, and low production cost. In this article, in addition to a brief overview of the structure and characteristics of nanobodies, the use of this molecule in the targeted drug delivery of breast cancer has been reviewed.

## Background

Despite various developments in breast cancer treatment, the survival rate of patients annually increased. Recent oncology studies present the new case, and the death rate of breast cancer has surpassed that of other more prevalent cancers [1]. The main molecular subtypes of breast cancer based on cell biomarkers, including Luminal A, Luminal B, HER2-positive, and triple-negative, can be used for therapeutic purposes. The more targeted therapy in breast cancer is designed on the immunohistochemical expression of hormone receptors: human epidermal growth factor receptor positive (HER2 +), estrogen receptor positive (ER +), and progesterone receptor positive (PR +) [2].

Some drawbacks of common chemotherapy and radiotherapy, especially off-target effects on cancer-free cells, reduce the quality of life and treatment efficiency of patients [3]. Also, the naked drugs and toxins for cancer treatment suffer from non-target toxicity and high-dose chemotherapy [4]. To overcome these limitations, monoclonal antibodies (mAb) are the superior biological moiety for diagnosing and targeting cancer, leading directly to target tumor cells along with the induction of long-lasting anti-tumor immune responses without damaging the health cells. On the other hand, intact mAb supplies antibody-dependent cell-mediated cytotoxicity via NK cells and tumor-associated macrophages [5, 6]. Although mAbs depend on the target, their relatively large size (150 kDa), slow tissue penetration, not binding to albumin, and pricey nature are their main limitations [7, 8]. To address these challenges, nanobodies (Nbs) are emerging as a new generation of cancer diagnosis and therapeutic approaches. Nbs derived from nurse sharks, spotted ratfish, and camel species are single-chain VHH antibody fragments with high stability that lack VL domains [9, 10]. Nbs are immunoglobulin-based recombinant antigen-binding proteins generated by immunizing the respective animal with the antigen of interest or an existing naïve library [11]. Nbs provide an excellent diagnosis and therapy toolkit with appropriate features such as a unique structure, a size 10 times smaller than traditional antibodies (~ 15 kDa), moderate cost, excellent periphery of tissue penetration, easy processing or modification, and the capability to bind albumin to enhance their lifetime [7, 12]. To advance the productive and precise elimination of cancer cells, high-specific antibodies and anticancer drugs in the form of antibody–drug conjugate (ADC) have been combined, which has become the best development of an anticancer drug delivery carrier [13]. Given the rapid rise in anti-cancer drugs with high efficacy since 2000, ADCs are called biological missiles [14]. The subsequent use of ADCs to improve cancer therapy, nanobody drug conjugates (NDC), is developing. NDCs, as a novel concept, was conceived as a positive cooperation by both Nbs and cytotoxic drugs for the progress of the therapeutic window. NDCs contain a tumor-targeting nanobody coupled to a toxic agent payload by a chemical linker [15]. Therefore, nanobodies are capable of binding to drugs and finally guiding cancer cells to the apoptosis pathway. Amidst the existing reports, we reviewed the structural design of nanobodies, nanobody generation methods, chemical procedures for nanobody conjugation with drugs, and various linkers to attach drugs to nanobodies, the recent progress of NDCs in breast cancer treatment, and briefly discussed the advance in the development of NDC. During our discussion, we hint at the challenges and opportunities of NDCs for breast cancer therapy.

### Structure, characterization and generation of nanobody

**Fig. 1:**
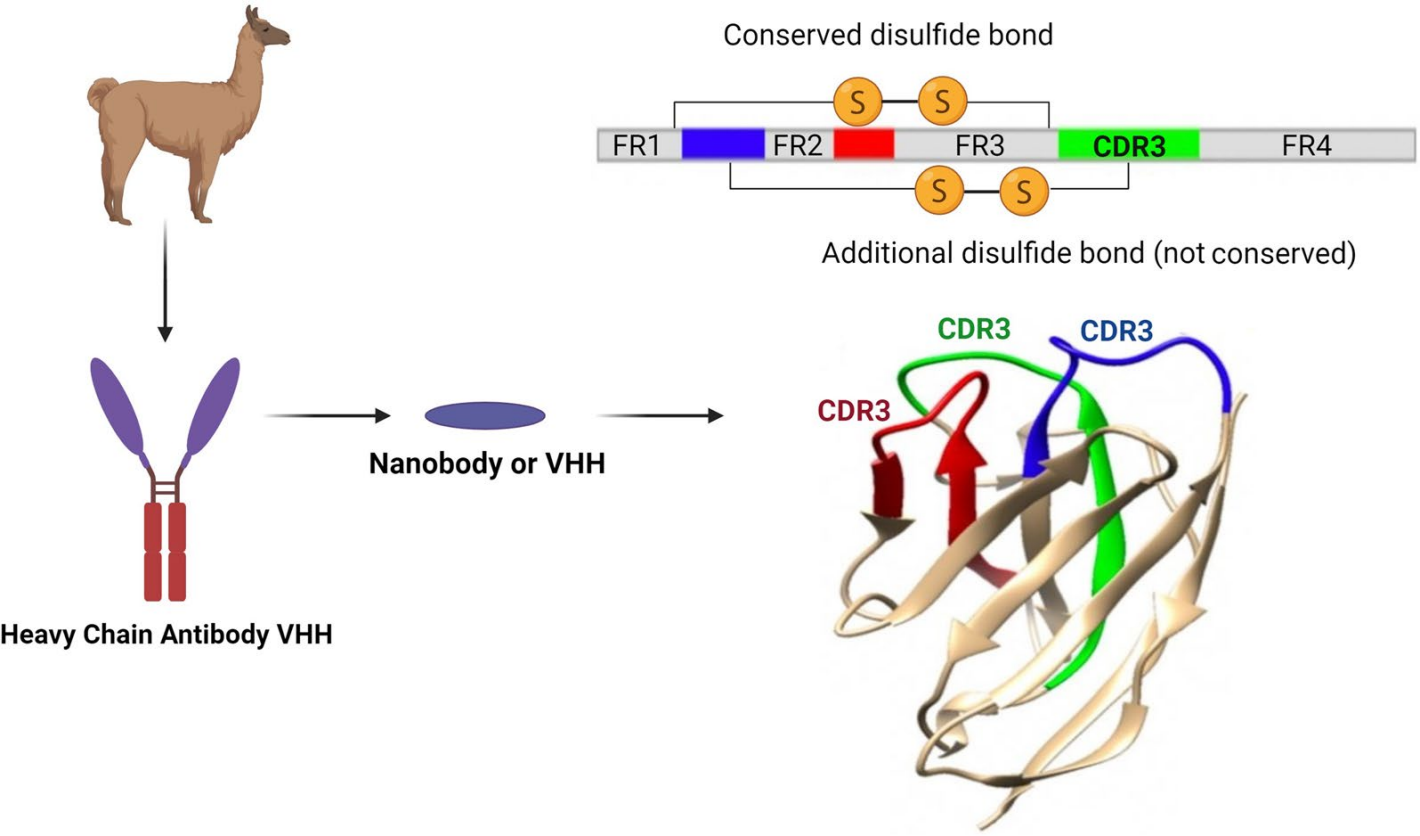
Nanobody, or VHH, is the variable segment of camel heavy chain antibodies, which consists of 4 conserved sequence segments (FR1/2/3/4) and 3 highly variable loops (complementarity-determining regions, CDR1/2/3) located at the tip of Nb. The CDR part of these nanobodies is wider than conventional antibodies and plays an important role in antigen binding

Nbs and the VH domains of Abs show significant architectural similarities. Specifically, Nbs are made up of 4 conserved sequence sections (FR1/2/3/4) and 3 highly variable loops (complementary determining regions, CDR1/2/3) located at the tip of the Nb (Fig. 1). The antigen-binding site of the Nb, also known as the paratope, is made up of three CDR loops [16]. CDR3 contributes the majority of the antigen-binding specificity, while CDR1 and CDR2 are in charge of boosting the binding strength [17, 18]. The longer CDR3 loop than that of ordinary Abs is one distinguishing feature of Nbs. While CDR3 loops in Abs only have 12 or 14 amino acids, the majority of Nbs’ finger-like CDR3 structures contain about 18 amino acids [19, 20]. Expanded CDR3 sections enable additional interactions between cognate antigen and Nb, compensating for the absence of the VL domain [21]. Nbs identify and interact with distinct epitopes through protrusion of CDR3, increasing potential targets [22].

Nbs’ small size makes them ideal for molecular imaging and solid tumor treatment due to their rapid diffusion and penetration. It is true to say that the small size of Nbs may also be a constraint, even if it is sometimes seen as a benefit, especially in non-invasive imaging [23]. Since Nbs’ molecular weight is below the threshold (50–60 kDa) for kidney glomerular filtration, the kidney’s quick removal of Nbs from the bloodstream could be problematic [24].

Frequent administration is required to achieve optimal efficacy because only a small portion of administered Nbs accumulates at the diseased sites. To overcome this limitation, significant efforts have been made to extend Nbs’ half-life [25]. It has been demonstrated that albumin can be used as a flexible platform to extend the half-life of drugs [26]. One work that used a bivalent EGFR-targeting and an albumin-targeting Nb to create a trivalent bispecific Nb was published in 2011 [27]. In mice, the trivalent Nbs had a prolonged half-life of 2–3 days due to albumin’s participation. As an alternative, Harmsen et al. found that when porcine IgG (pIgG) was attached to Nbs, the in vivo residence period was 100 times longer than it was for control Nbs, which are incapable of binding to pIgG [28]. The exceptional resilience of Nbs is another trait. It has been shown that four hydrophilic amino acids are replaced with four hydrophobic amino acids in the FR2 region of conventional Abs. It has been proposed that the hydrophobic amino acids on the surface of the VH domain in typical Abs serve as the binding surface for VL domains. The fundamental cause of Nbs’ improved solubility and stability has been attributed to its characteristic substitution of hydrophobic amino acids with hydrophilic amino acids [21]. The primary cause of the poor immunogenic profiles of Nbs is their high sequence identity. VHH exhibits a significant degree of sequence identity with the human type 3 VH domain, according to published sequence information [29]. The non-human origin of nanobodies may cause unwanted immune responses and ADA (anti-drug antibody) production when administered to humans. However, the induction of immune responses is more likely when nanobodies are aggregated. Amorphous aggregates of VHHs can stimulate an undesirable immune response, such as ADA. Golam Kibria et al. examine the immunogenicity of an anti-EGFR nanobody in several types of aggregates. ELISA findings showed that the native nanobody was barely immunogenic, while VHH-65 (VHH incubated at 65 °C) was slightly immunogenic. Moreover, the misfolded aggregates and VHH-95 (VHH incubated at 95 °C) were extremely immunogenic. These findings showed the vital role of the physicochemical properties of nanoparticles in creating an immune response [30]. The low immunogenicity of VHHs has also been demonstrated in Phase I clinical trials conducted by Ablynx. Nbs are suitable for sustained and repeated administration due to their low immunogenicity. Additionally, methods for Nb humanization have been created and are now standard practice prior to clinical trials [31, 32], substantially reducing unfavorable immunological reactions. It should be highlighted, though, that more engineering may be required after humanization in order to restore Nbs' adequate binding capacity for their targets [3]. Nbs may be expressed in large quantities utilizing inexpensive production systems like *E. coli* and *S. cerevisiae* because of their hydrophilicity, lack of posttranslational modification, fewer disulfide linkages, and monomeric structure. Contrastingly, the manufacture of scFv frequently has a low yield due to its lowered stability and solubility [33].

### Nanobody drug conjugation (NDs)

**Fig. 2:**
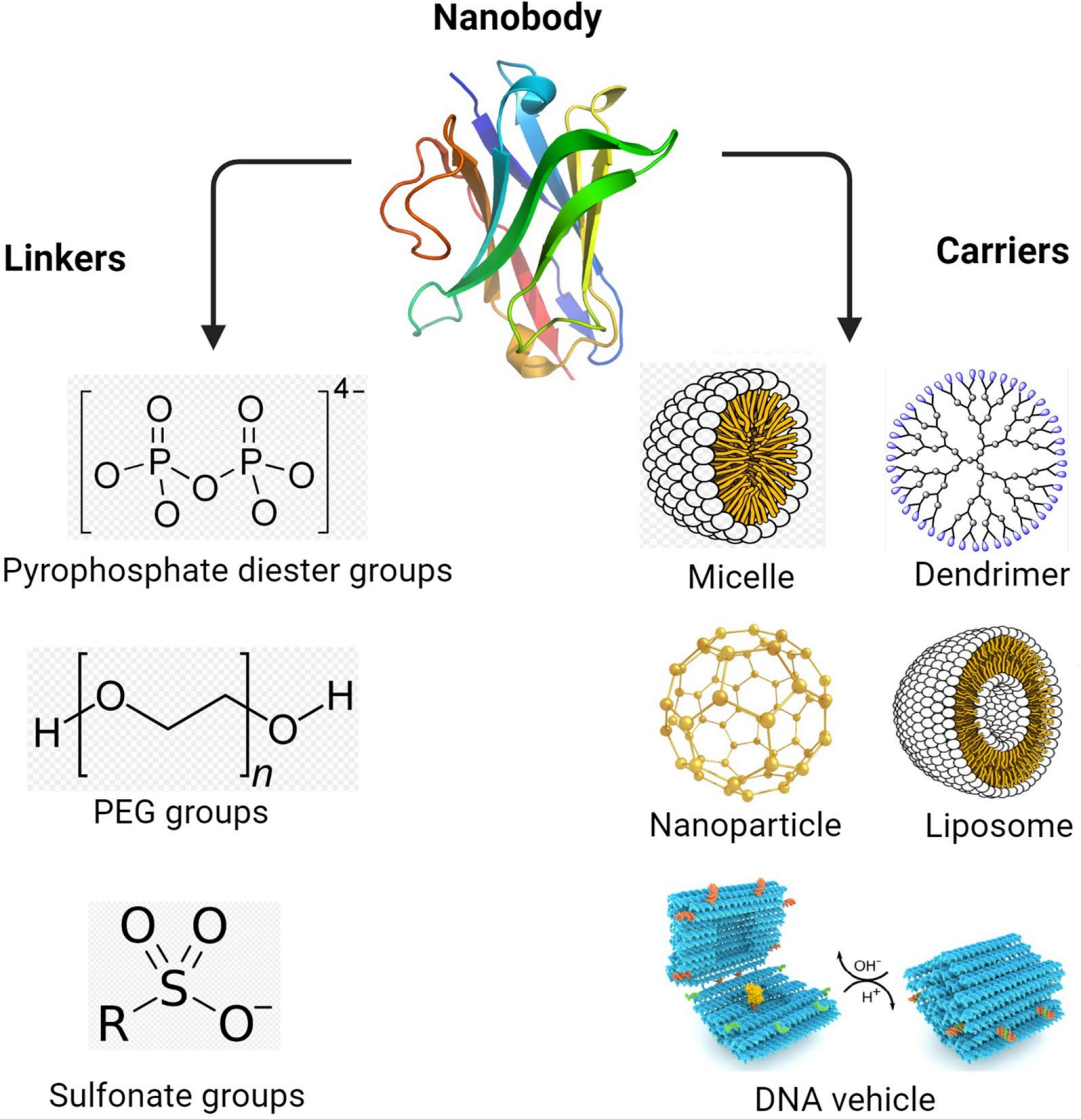
Nanobody can be conjugated with carrier or vehicle molecules through chemical linkers. Next, the desired molecules can be specifically transferred to the cancer cells through the carrier or vehicle

Global concerns have been raised about chemotherapy-related cytotoxic side effects and drug resistance in patients. The research on nanobodies (Nb) offers the possibility of developing nanotechnology-based tools for targeted drug delivery [34] and ultrasensitive sensors for personalized treatment [35, 36]. All such tools would aim to reduce chemotherapy side effects. It is still necessary to research ADCs further in order to overcome the drawbacks associated with targeted chemotherapy. NDCs with chemo drugs offer a cheaper, more stable, and highly specific alternative with low production costs and long-term stability. Chemo drugs can be conjugated with NDCs through chemical linkers to form carrier or vehicle molecules (Fig. 2). To enhance their efficiency, some factors, including chemo drug type, site-specific attachment strategy, specificity, affinity, chemo drug/Nbs ratio, and chemo drug location, must be considered. NDCs target cancer sites and recognize cancer antigens through receptor-mediated endocytosis, releasing activated chemo drugs into the cytoplasm, which inhibits cell proliferation, leading to cell apoptosis [34].

In a recent study by Xu et al., a novel nanobody drug conjugate (NDC) was developed for TROP2-positive pancreatic cancer treatment. TROP2 (trophoblast cell surface antigen 2) is a cell surface antigen that is highly expressed in pancreatic tumor cells. Most of the targeted ADCs that have been developed against TROP2 are of the IgG type and have performed poorly. The NDC, Nb-TROP2-HAS-MMAE, consists of a nanobody against TROP2 and human serum albumin (HSA), which, after intracellular transfer to lysosomes, releases MMAE in the cell and induces cell apoptosis in TROP2-positive pancreatic cancer cells. Interestingly, this conjugated nanobody showed significant antitumor effects in the pancreatic cancer xenograft model (doses of 0.2 mg/kg) and was able to eradicate the tumor at high doses (5 mg/kg). As a result, the use of NDCs as a potential treatment option for cancer treatment is very promising [37].

In another study, Liu and colleagues designed a novel nanobody-ferritin platform for targeted drug delivery in EGFR-positive cancer cells. As a protein nanocage, ferritin has a hollow cavity that can create an enclosed environment for various therapeutic agents. The combination of ferritin with nanobody leads to the production of a molecule that not only protects the drug, but also has the ability to identify specific cells. Conjugation between ferritin and the nanobody is achieved using transglutaminase. It should be noted that the conjugation of Nb to ferritin is done after loading the drug (manganese phthalocyanine, a photodynamic reagent) in ferritin, because the harsh conditions of encapsulating the drug in ferritin (harsh pH) can cause the inactivation of the nanobody. This nanobody conjugate (MnPc@Nb-Ftn) can effectively enter EGFR-positive A431 cancer cells, while it has no effect on EGFR-negative cells (MCF-7). Using a laser with a wavelength of 730 nm, MnPc@Nb-Ftn selectively induces the production of ROS (reactive oxygen species) in A431 cells and destroys the cells [38].

### Linkers in NDs

The linker is crucial for maintaining Nb and chemo drug conjugation, ensuring enhanced stability and prolonged blood circulation in NDCs. Ideal linkers must meet high stability in human blood plasma, hydrophobicity to minimize side effects, and self-cleavage capacity in specific environments unique to tumors [39]. In fact, inefficient binding may lead to premature drug release from nanobodies before reaching the target tumor site, leading to adverse chemotherapeutic side effects [39]. Another important challenge is the degree of linker hydrophobicity. Attachment of hydrophobic chemical drugs to hydrophobic linkers can increase aggregation, which may result in renal clearance or trigger unwanted immune responses. Hydrophilic linkers, such as pyrophosphate diester groups [40], polyethylene glycol (PEG) groups [41], or sulfonate groups [42], can reduce aggregation and increase the chemo drug to Nb ratio. Cleavable or non-cleavable linkers are essential for maintaining NDC systemic circulation and releasing payloads after reaching the target site, as well as site-specific conjugation [43]. In the study of Li et al., the use of PEG chains as linkers in nanobody conjugates significantly increased the half-life of circulation and also clearly reduced the cytotoxicity of conjugates. In this study, a new design strategy for NDCs was proposed, in which drug molecules and nanobodies are specifically coupled through a bifunctional PEG (polyethylene glycol) chain. The outcomes exhibited that compared to VHH-SMCC-MMAE conjugate, which has no PEG insert, VHH-PEG10K-MMAE, and VHH-PEG4K-MMAE, they have 11.2 and 2.5 times increased half-lives and 22 and 4.5 times reduced cytotoxicity, respectively. The results showed that increasing the half-life is very useful in improving the therapeutic capacity of NDCs [44].

### Site specific conjugation strategy

Chemical conjugation of Nbs with chemotherapy drugs has extended their applicability and improved clinical outcomes [45]. Modifications focus on utilizing amino acids found naturally on Nbs' protein backbones, such as cysteine, lysine, aspartic acid, and glutamic acid. This can lead to higher drug-to-Nb ratios but may also create heterogeneous mixtures, potentially affecting the overall efficacy of NDCs. Advances in protein chemistry have enabled new tools to control conjugation and form homogenous NDC mixtures [46, 47]. Although new groups of anti-cancer medicines are starting to emerge, the chemotherapy drugs or other anti-cancer compounds used in developing NDCs can be broadly divided into two classes: microtubule inhibitors and DNA-damaging agents [48].

One of the most important steps in creating reliable probes for immuno-detection applications is labeling antibodies and their fragments (nanobodies, single-chain Fv, etc.) uniformly. Many site-specific coupling techniques based on chemistry, genetics, and peptides have been developed to date. Co-assembling peptide tags is one of these techniques that is the easiest to use and most adaptable. In a study, Moeglin et al. performed site-specific labeling of VHHs using two related peptide tags, K3 and E3, which originate from the p53 tetramerization domain. The K3 and E3 tags provide a reliable and easy technique to connect stoichiometric levels of VHH and fluorescent probes at specific sites. A nanobody targeting HER2 is genetically fused to E3 and linked to various fluorescent derivatives of K3. These molecular probes exhibit great binding selectivity on HER2-overexpressing cells in flow cytometry. Additionally, they are sufficiently durable to stain the HER2-receptor on living cells, which may be seen using fluorescent confocal imaging [49]. Considering the easy production of recombinant nanobodies in microorganisms, the use of this group of binders in the field of molecular imaging is attractive. Due to more uniform protein-to-fluorophore stoichiometry, less background staining, and a known distance between dye and epitope, site-specific fluorescent labeling of nanobodies can further improve the effective spatial resolution in single-molecule localization microscopy techniques. For this reason, Teodori et al. describe in a study a methodology for site-specific bio-conjugation of DNA oligonucleotides to three different VHH produced with a C- or N-terminal unnatural amino acid, 4-azido-L-phenylalanine. The VHH-oligonucleotide conjugation procedures were effective and produced very pure bio-conjugates using copper-free click chemistry. Target binding was preserved in the bio-conjugates, as shown by DNA points' accumulation for imaging in nanoscale topography. This site-specific oligonucleotide-protein conjugation technique can be expanded for use in molecular targeting and drug delivery applications where stoichiometric control and site-specificity are necessary [50].

### Nanobody based drug delivery (NDv)

Drug delivery based on nanobody can be studied in several categories. In the first category, the binding of toxic agent or drug to nanobodies is investigated. In the second part, the conjunction of nanocarriers (liposome, nanoparticle, etc.) to nanobodies is explained. In this section, drugs and chemotherapy agents are accommodated in nanostructures and then connected to nanobodies or VHHs. The attachment of radioactive substances and nanobody radio labeling with will be examined in the third part.

#### Nanobodies attached to the drug and toxic molecule

One of the proteins that has a high expression in tumors, including breast cancer, is CD147. This protein stimulates cancer cell growth, metastasis, and invasion. The attractive features of nanobodies and the absence of nanobodies against CD147 led Li et al. to design and produce a nanobody against CD147 (VHH-11-1) using the phage display technique. In the following, this nanobody was successfully conjugated with doxorubicin (DOX). The chemical coupling approach was successfully used to manufacture DOX-11-1, which showed potential anticancer effects in both in vivo tests and in vitro cells expressing high levels of CD147. In general, 11-1 is a potential Nb that will offer a unique approach to the treatment of malignancies with high levels of CD147 expression, and successfully has a synergistic effect on inhibiting 4T1-bearing mice when combined with doxorubicin. Tumor targeting and antitumor effects were found in CD147-positive tumors in vitro and in vivo. Considering the synergistic effect of nanobody and doxorubicin in inhibiting cancer in 4T1 carrier mice, this method may provide a new guide for breast cancer treatment [51].

Photodynamic therapy (PDT) using nanobodies targeting human epidermal growth factor receptor 2 (HER2) is a promising treatment for a significant number of breast cancer patients with residual disease after neoadjuvant or trastuzumab resistance. In the study of Daken et al., the antitumor efficacy of a HER2-targeted nanobody photosensitizer (PS) conjugated to IRDye700DX was investigated in vitro and in vivo. The HER2-targeting nanobodies were obtained via phage display and engineered into a biparatopic construct. HER2-targeted nanobodies were conjugated to a photosensitizer and evaluated by in vitro PDT assays. The results showed targeted tumor cells with 1D5-PS and 1D5-18A12-PS induced significant tumor regression in trastuzumab-resistant high-HER2-expressing tumors, whereas in low-HER2-expressing tumors only a slight growth delay was observed. Moreover, mice with trastuzumab-resistant tumors were treated with nanobody-PS conjugates. The tumors were illuminated with a fluence (100 J∙cm-2 at 50 mW∙cm-2) after nanobody-PS administration, and the tumor growth was measured for 30 days. The selected nanobodies remained functional after conjugation to the PS, with high affinity for HER2-positive cells. Both nanobody-PS conjugated potently and selectively induced cell death of HER2 overexpressing cells, either sensitive or resistant to trastuzumab, with low nanomolar LD50 values. This research suggests a new way to treat HER2-positive breast cancer by using nanobody-targeted PDT as a new adjunctive therapy, especially for trastuzumab-resistant patients. Interestingly, they selectively induced tumor regression in HER2 tumors in one session. Moreover, these nanobody-PS conjugates could be used for optical imaging in the cancer detection field [52].

Inflammatory factors such as cytokines in the tumor environment can promote cancer progression. Targeting cytokines may be important for treating metastatic breast cancer. For example, neutralizing harmful TNF signaling with an anti-TNF-α antibody can inhibit cancer metastasis and invasion. Ji and colleagues illustrated the significance of neutralizing TNF-α levels in the tumor microenvironment to enhance chemotherapy response, with promising clinical implications. In their study, the high-affinity (2.05 nM) TNF-α-specific VHH was produced using nanobody technology in a *Pichia pastoris* host. The TNF-α nanobody inhibited breast cancer cell proliferation induced by human TNF-α in a dose-dependent manner. Moreover, this nanobody prevented the invasiveness of MDA-MB-231 and the migration of MCF-7 and MDA-MB-231 induced by human TNF-α. Combining paclitaxel with TNF-α nanobody improved efficacy against breast tumor proliferation and lung metastasis. Considering that neutralizing low levels of cytokines such as TNF-α in the tumor microenvironment has positive results in cancer treatment, the use of nanobodies to control cytokines and also drug delivery in these environments has an attractive potential for clinical applications [53].

In a different investigation, Ji and associates created an innovative hybrid nanobody to enhance the anticancer efficacy of the anti-TNFα nanobody in triple-negative breast cancer. In this study, an anti-TNFα nanobody was bonded to the RGD4C peptide to examine the anti-cancer activity in vitro and in vivo. The RGD4C peptide comprises the RGD sequence, the smallest recognition part that binds to the αvβ3 receptor on the membranes of tumor cells and is implicated in the metastasis, proliferation, and adhesion of tumor cells. V-L-R-H has the ability to bind to αvβ3 and effectively inhibit MDA-MB-231 cell proliferation and migration. This fusion nanobody (V-L-R-H) was evaluated for anti-cancer activity in a xenograft mouse model and could inhibit tumor metastasis and proliferation. Also, this nanobody is capable of inhibiting the process of EMT (epithelial-mesenchymal transition) by reducing Ki67, HIFα, CD31, and TNFα. Considering that TNBC is not sensitive to current therapy, new treatment methods can be helpful. This bifunctional fusion protein against TNFα and αvβ3 can provide a novel research direction for TNBC treatment [54].

One of the ways to fight BLBC (basal-like breast cancer) that causes brain metastasis (BM) is to target DR4/5 (death receptor) and EGFR antigens, which are overexpressed in BLBC. Nevertheless, drug delivery to these targets is difficult due to the location of brain metastasis. In a study by Kitamura and co-workers, BLBC with brain metastasis mouse models were developed with different patterns of metastasis. In the following, the adaptability of stem cells mediated by bi-functional DR4/5 and EGFR-targeted therapy was investigated. Most BLBC lines were sensitive to E_V_DR_L_, a bi-targeting therapeutic protein that combines anti-EGFR VHH (E_V_) with DR ligand (DR_L_). Functional analyses showed that the E_V_ domain would be effective in enhancing DR4/5-DR_L_ binding and increasing DR_L_-induced apoptosis. Stem cells that secrete E_V_DR_L_ reduce tumor burden and increase survival in animal models. As a result, co-targeting DR4/5 and EGFR in BLBC showed to be effective in treating brain metastasis in breast cancer [55]. Table 1 briefly shows the nanobodies of this parts.

**Table 1:** Studies conducted in the field of targeted drug delivery by nanobody with direct connection to drug and toxic molecule

Name of NDs	Antigen	Type of drug or other molecule	Type of study	The mechanism of action and application	References
DOX-11-1	CD147	Doxorubicin	Pre-clinical/ Balb/c mice bearing 4T1 tumor cells	Antitumor activities by blocking topo-isomerase 2	Li et al. [51]
1D5-PS 1D5-18A12-PS	HER2	IRDye700DX	Pre-clinical/ Balb/c mice bearing HCC1954 tumor cells	Regression of high HER2 expressing tumors	Deken et al. [52]
TNF-α nanobody	TNF-α	Paclitaxel	Pre-clinical/ Balb/c mice bearing 4T1 tumor cells	Neutralizing low levels of TNF-α in the tumor microenvironment that inhibit tumor proliferation and metastasis	Ji et al. [53]
V-L-R-H	TNF-α	RGD4C peptide	Pre-clinical/ Balb/c mice bearing MDA-MB-231 tumor cells	Inhibiting the process of EMT (epithelial-mesenchymal transition) by reducing Ki67, HIFα, CD31, and TNFα	Ji et al. [54]
E_V_DR_L_	EGFR	Death receptors 4/5 ligand (DRL)	Pre-clinical/ Mouse model	Treating brain metastasis in breast cancer	Kitamura et al. [55]
VHH-PEG-PEI	HER2	PEGylated polyethyleneimine	Pre-clinical/ BT474, SKBR3, NIH3T3, and MCF10A cell lines	VHH conjugated by PEG-PEI copolymers might be an efficient nanocarrier for specific targeting of Her2 positive tumors	Saqafi et al. [56]

The in vivo application of polyethyleneimine (PEI), as one of the most efficient non-viral gene transfer agents, is limited due to some problems such as relatively high cytotoxicity, interaction with blood components, and non-specific binding to cells. In a study by Saqafi et al., a nano-carrier system based on PEI derivatives was designed through the attachment of a branched polyethyleneimine polymer (25 kDa) to MAL-PEG3500-NHS (bi-functional polyethylene glycol molecules). This compound was made with three different molar ratios of 10, 20, and 30 (polyethylene glycol: polyethylene imine). The anti-HER2 nanobody as a targeting agent was conjugated to the copolymer made of polyethylene glycol and PEI as a targeting agent. In the following, the effect of these changes on the gene transfer efficiency, cytotoxicity, physicochemical properties, and cell absorption of PEI polymers was assessed. PEGylated PEI copolymers were less cytotoxic in vitro than unconjugated PEI. Also, the PEG-PEI copolymer conjugate (10:1 molar ratio) exhibited low cytotoxicity and favorable physicochemical properties. On the other hand, the combination of the anti-HER2 nanobody with PEI decreased the toxicity of this substance in all cell lines (BT-474, SK-Br-3, NIH3T3, and MCF-10A cell lines) studied by 1.5–2 times. Moreover, transfection ability and cellular uptake of this copolymer were improved in HER2-positive breast cancer cell lines, suggesting potential for targeted therapy [56]. All the nanobodies in this section (Table 1) are currently in the pre-clinical phase and have not yet entered the clinical trial phases.

#### Nanobodies connected to nanocarriers

The simultaneous use of several antibodies that target different epitopes of a specific tumor antigen is a successful platform for cancer treatment. Increasing the drug binding capacity and improving the cytotoxicity of malignant cells are the advantages of active targeting of liposomal drugs. Farasat and colleagues represented a promising strategy for managing HER2-overexpressing breast cancers through the utilization of nanobody-bound liposomes to target cancer cells. In their study, four anti-HER2 recombinant VHHs were produced and purified in a native form. A combination of four VHHs was utilized to specifically target HER2-overexpressing cells. These nanobodies were conjugated with PEGylated liposomes containing doxorubicin. The amount of drug encapsulation was reported to be about 94%. In this study, targeted liposomes were effectively bound to SKBR3 and BT474 cell lines as HER2-positive cells. Oligoclonal VHH mixture-conjugated liposomes exhibit superior binding efficiency compared to the monoclonal version. Moreover, oligoclonal nanoparticles were more toxic to HER2 + cells than non-targeted liposomes [57].

The potential applications of nanoparticles in personalized medicine are varied, including drug delivery and the ability to monitor cancer treatment in real time. The objective of the study conducted by Khaleghi et al. was to develop a targeted immunoliposome labeled with fluorescent markers to facilitate the visualization of drug delivery and distribution in real-time. In their study, fluorescent liposomes labeled with anti-HER2 VHH improved drug delivery monitoring and tumor cell tracking with few side effects. They detected nanoparticles in the target cells by confocal microscopy. The results indicated the intracellular absorption of 110 ± 10 nm particles with a conjugation efficiency of nearly 70%. Moreover, live-cell trafficking was checked using microscopy during incubation, and according to the results, fluorescent-labeled nanoparticles bonded to the HER2-positive breast cancer cells with negligible off-target. By using the combination of nanobodies and nanoparticles, it is possible to make significant progress in the treatment of breast cancer by delivering specific and real-time drugs. Also, this method can be used for targeted delivery of contrast materials in thermotherapy and imaging [58].

In a study by Banihashemi et al., the liposome nanoparticle was prepared using classical lipid film formation, then conjugated to anti-CD19 VHH. LF (Lethal Factor) was loaded into anti-CD19 liposome nanoparticles to block MAPK signaling in Raji B cells. LF is a part of Bacillus anthracis LT (lethal toxin), which is a MAPK inhibitor. MAPK is a vital signaling pathway in cancer development and metastasis. The targeted cytotoxicity of the LF immunoliposome was confirmed by the BrdU lymphoproliferation assay. Liposome nano-formulation was optimized to reach maximum LF encapsulation and targeted delivery. Next, phosphorylation of MAPK pathway mediators like MEK1/2, P38, and JNK was inhibited following the treatment of Raji cells with LF-immunoliposome. They assessed the effect of formulation on pro-apoptotic genes, and the result showed upregulated of caspase genes, clearly illustrating cell death induced by LF through pyroptosis and caspase-dependent apoptosis. This VHH immunoliposome has a potent MAPK inhibitor targeting B cells, which curbs proliferation and ushers B cells toward apoptosis. Interestingly, this anti-CD19 immunoliposome targeted CD19 + cancer cells while keeping normal cells intact [59].

In another study, Khodabakhsh et al. evaluated the enhancing antibody targeting by conjugating anti-MUC1 VHH to the chitosan nanoparticles. The synthesis of chitosan nanoparticles (8- 10 kDa) was carried out using the ionic gelation technique, and the NHS-EDC method was used to attach the chitosan to the VHH. The MTT assay showed that the anti-MUC1 conjugated by nanochitosan was more effective than the free anti-MUC1 nanobody in inhibiting breast cancer cell (MCF-7) growth. Additionally, chitosan nanoparticles were not cytotoxic to MCF10 cells. As a result, chitosan nanoparticles may be ideal for delivering MUC1 nanobodies to breast cancer cells with high MUC1 levels and decreasing the required dose [60].

Polymeric nanoparticles can deliver cytotoxic proteins such as saporin, which lack defined mechanisms to enter the cell, to cancer cells. In the study by Martinez-Jothar et al., PEGylated poly “lactic acid-co-glycolic acid-co-hydroxymethyl glycolic acid” (LGHMGA) nanoparticles were used to deliver saporin to the cytoplasm of the HER2-positive breast cancer cells. Binding of nanoparticles to 11A4 nanobody (a specific VHH against the HER2 receptor) enabled rapid and extensive selective uptake of nanoparticles for HER2-positive cells (SkBr3), while in HER2-negative cells (MDA-MB-231) there was no absorption of nanoparticles. Interestingly, this selective uptake is blocked if the cells are incubated with an excess amount of nanobodies. Moreover, Cys-NPs, as nontargeted nanoparticles, were not taken up by the cells. A dose-dependent cytotoxic effect was only seen on SkBr3 cells when treated with saporin-loaded 11A4 nanoparticles in combination with PCI (photochemical internalization). This combination induced apoptosis, strongly decreased viability, and repressed cell proliferation. Excess nanobodies reduced the cytotoxicity of saporin-loaded NPs and enhanced selectivity. According to the result, the combination of targeting VHH on nanoparticles with cytotoxic drugs is an effective tool to achieve selective uptake of these nanoparticles [61].

Although HER2 is overexpressed in 20–25% of breast cancers, the hiding of many important epitopes of this antigen due to genetic drifts has made targeting HER2 an important challenge. Nikkhoi and colleagues, et al., developed bivalent bispecific, bivalent monospecific, and monovalent nanobodies. Among these, bivalent bispecific had the highest affinity towards HER2 + cells compared to others. VHHs were coupled with fluorescently labeled liposomal nanoparticles to form targeted liposomes. The bivalent, bispecific VHH focused on liposomes had the highest fluorescent intensity on HER2 breast cancer cells (BT-474 and SKBR-3 cell lines), according to flow cytometry results. In addition, liposomes conjugated with a bivalent monospecific nanobody showed a higher affinity for HER2-positive cell lines compared to monovalently targeted liposomes [62].

In another study, Nikkhoi and colleagues produced four anti-HER2 nanobodies in the prokaryotic host cell, which yielded disulfide-bonded nanobodies. The purified nanobodies were analyzed pre- and post-thiolation through Western blot, and their effectiveness against the HER2 receptor ectodomain was confirmed via flow cytometry and ELISA. Then, thiolated nanobodies were bound to reactive DSPE-PEG (2000) maleimide incorporated into the vesicles. The tetra-specific multivalent methotrexate-loaded nanobody PEGylated liposomes appeared to have more binding avidity and cytotoxicity in HER2-overexpressing breast cancer cells (BT-474 and SKBR-3 cell lines) compared to the top-acting monoclonal nanobody vesicles. This study concerned the effective approach for designing and engineering anticancer nanomedicines utilizing VHH expression for future applications in personalized and exact treatments and diagnostics [63].

In a study by Sayed-Tabatabaei et al., co-delivery of nitroxoline (NIT) and cisplatin (CIS) using targeted liposomes was done by anti-HER2 VHH. NIT is an anticancer antibiotic that inhibits cell migration by blocking the cathepsin B enzyme, and CIS is used to treat various malignancies, including metastatic breast cancer. The aim of this study was to increase toxicity and inhibit cell migration of cancer cells, specifically through targeted liposomes conjugated with anti-HER2 nanobodies. Production of liposomes was performed by the solvent injection technique using Mal-PEG-DSPE (maleimide-polyethylene glycol 2000 distearoyl phosphatidyl ethanolamine), soy lecithin, and cholesterol. In the following, liposomes were specific by binding to specific anti-HER2 nanobodies for targeting metastatic breast cancer cells. Next, optimization of liposomes was done for their physicochemical characteristics such as release efficiency, zeta potential, particle size, and drug loading. The effects of targeted liposomes loaded with NIT-CIS on TUBO and MDA-MB-213 cell lines as HER2 + and HER2-, respectively, were investigated in cellular processes including apoptosis, cytotoxicity, cell uptake, and migration. Findings showed that the combination of NIT and CIS increased cytotoxicity meaningfully. Furthermore, examining cell migration showed that NIT compounds play an effective role in reducing the invasiveness of cells. Also, these targeted liposomes enhanced cellular uptake and cytotoxicity in HER2-positive cell lines, while this did not happen in HER2-negative cells. Finally, the antitumor activity of targeted liposomes containing CIS-NIT was performed in vivo with free drugs and non-targeted liposomes. The results showed that the targeted liposomes containing CIS-NIT were able to suppress breast cancer caused by TUBO cells in Balb-c mice with extraordinary efficiency compared to the control group [64].

Wang et al. developed a QD (quantum dots)-based micelle loaded with AF (aminoflavone) as an anticancer drug that was conjugated with a specific anti-EGFR nanobody. This nanopharmaceutical serves as a theranostic nanoplatform for EGFR-overexpressing cancers, including TNBCs (triple-negative breast cancer). In this study, researchers used the near-infrared fluorescence of the InP/ZnS (indium phosphate core/zinc sulfide shell) QDs for in vivo biodistribution studies of nanoparticles. On the other hand, using 7D12 nanobody conjugated to QD-based micelles enhanced uptake and cytotoxicity in EGFR-overexpressing TNBC cells. Targeted micelles containing AF (conjugated with nanobody) had higher accumulation in tumors than non-targeted micelles containing AF (without Nb conjugation), which led to more effective tumor regression in a breast cancer mouse model. Considering that no systemic toxicity was observed with the treatments, it can be concluded that this QD-based nanobody-conjugated micelle can help as an effective theranostic platform for cancers such as TNBC [65].

Zou and colleagues made and characterized polymersomes conjugated with VHHs by utilizing FDA-approved, biodegradable, and biocompatible PEG-b-PCL. Mal and FITC (fluorescein isothiocyanate) functionalized copolymers such as FITC-PEG-b-PCL and Mal-PEG-b-PCL were created by reacting FITC and N-beta-maleimidopropyl-oxysuccinimide ester with H2N-PEG-b-PCL. The preparation of polymersomes was done using a mixture of FITC-PEG-b-PCL, Mal-PEG-b-PCL, and MeO-PEG-b-PCL through thin film hydration and nanoprecipitation techniques. Cryogenic TEM revealed vesicular structures (polymersomes) for nanoparticles. The following mean diameters (~ 150 nm) and zeta-potentials (~ − 1 mV at pH 7.4) were assessed by the DLS (dynamic light scattering) method. Nanoparticles were functionalized with anti-GFP or anti-HER2 nanobodies via maleimide-cysteine, increasing their zeta potential and size. Nanobody-conjugated polymersomes and non-targeted nanoparticles were tested for binding to HER2-positive breast cancer cells utilizing confocal microscopy and flow cytometry, and the results illustrated that anti-HER2-VHH-functionalized PEO-b-PCL polymersomes target HER2-overexpressing breast cancer cell lines. Comprehensive morphological studies and cell attachment resulting from this study open the way for other investigations to evaluate the possibility of using these nanoparticles for targeted drug delivery [66]. Table 2 briefly shows the nanobodies that are attached to a nanocarrier, and the desired drug is enclosed in the nanopart. The nanobodies in this part are currently in the pre-clinical stage and have not yet entered the clinical trial stages.

**Table 2:** Studies conducted in the field of targeted drug delivery by nanobody

Name of NDs	Antigen	Type of carrier/drug	Type of study	The mechanism of action	References
Oligoclonal HER2/Doxo	HER2	PEGylated liposomes /doxorubicin	Pre-clinical/ SKBR3 and BT474 cell lines	Stops the growth of cancer cells by blocking topo-isomerase 2	Farasat et al. [57]
Anti-HER2 VHH FL	Her-2	Liposome/fluorescent markers	Pre-clinical/ Her2 positive cell lines	Monitoring and tumor cell tracking	Khaleghi et al. [58]
Anti-CD19 VHH-LF	CD19	Liposomes nanoparticle/ Lethal Factor (LF) of Bacillus anthracis	Pre-clinical/ Raji B cell line	Block MAPK signaling, in the Raji B cells	Banihashemi et al. [59]
Anti-Muc1-VHH	MUC1	Chitosan	Pre-clinical/ MCF7 cell line	Inhibiting breast cancer cell growth	Khodabakhsh et al. [60]
11A4-NPs	HER2	PEGylated PLGHMGA nanoparticles/ saporin	Pre-clinical/ SKBR3 cell line	Ribosome inactivating protein	Martinez-Jothar et al. [61]
RR2-H-RR2-lip RR4-H-RR4-lip	HER2 HER2	Liposomes/lissamine rhodamine B sulfonyl	Pre-clinical/ BT474 and SKBR3 cell lines	Highest fluorescent intensity on HER2 breast cancer cells	Nikkhoi et al. [62]
oVHH-Lip	HER2	Liposomes /methotrexate	Pre-clinical/ BT-474 and SKBR-3 cell lines	Inhibits dihydrofolate reductase (DHFR) and prevent the tetrahydrofolate synthesis	Nikkhoi et al. [63]
11A4-CIS-NIT	HER2	Liposomes /Nitroxoline (NIT)-Cisplatin (CIS)	Pre-clinical/ TUBO cell line	NIT: inhibits cell migration by blocking cathepsin B enzyme CIS: is used to treat various malignancy including metastatic breast cancer	Sayed-Tabatabaei et al. [64]
7D12-QD-AF	EGFR	QD (quantum-dot) micelles/AF (aminoflavone)	Pre-clinical/ MDA-MB-468 cell line	Tumor regression in breast cancer through induction of oxidative DNA damage and reactive oxidative stress	Wang et al. [65]
VHH1-FITC-PS	HER2	PEG-b-PCL/ FITC or maleimide	Pre-clinical/ SKBR-3 cell	Use in studies to evaluate the use of nanoparticles for targeted drug delivery	Zou et al. [66]

#### Radiolabeled nanobodies

Due to their small size and high affinity, nanobodies have a high ability to penetrate tumor tissues. On the other hand, these proteins have a low biological half-life and are quickly cleared from the bloodstream. For this reason, they can be used as a useful tool in radioactive labeling. This allows diagnostic scans to be performed only a few hours after the injection of the tracer, and the ride-active substance is quickly eliminated from the body [67–69]. In several animal studies, less than 0.5% of the injected activity per gram of tissue was present in the blood one hour after injection [70, 71].

However, rapid clearance of nanoparticles from the blood can present another problem. The rapid clearing of the nanobody prevents the required circulation of the radiolabeled nanobody in the patient’s blood, and only a small part of the prescribed nanobody reaches its target. As a result, in order to obtain valid results, multiple doses of nano-body should be administered inside the body. Multiple injections also lead to kidney accumulation of nano-bodies, which is a major drawback of using radiolabeled nanobodies as in vivo imaging probes [72].

RPT, or radiopharmaceutical therapy, exhibits potential as a therapeutic modality for cancer types that demonstrate overexpression of HER2, such as breast cancer. Single-domain antibody fragments, such as VHH, are well-suited for RPT due to their rapid tumor accumulation and faster clearance from normal tissues compared to intact antibodies. In the study conducted by Feng et al., the VHH was 1028 subjected to labeling with iso-[131I] SGMIB (N-succinimidyl 3-guanidinomethyl 5-[131I] iodobenzoate). Next, its distribution was assessed in the xenograft mouse model of breast cancer expressing HER2 antigen (by the BT474 cell line). Comparing iso-[131] SGMIB-VHH_1028 with a HER2-targeted radiopharmaceutical (under clinical trial), the radiopharmaceutical in this study demonstrated an elevated level of tumor uptake and a reduced accumulation in the kidney. The findings of the study indicated elevation ratios (6.3 times) of tumor-to-kidney radiation doses in the xenograft model. This radiolabeled nanobody was subjected to purification through the solid-phase extraction process, bypassing the need for high-performance liquid chromatography purification. The findings of the present investigation demonstrate the potential of iso-[131I] SGMIB-VHH_1028 as a viable radiopharmaceutical therapy for malignancies that express HER2. In addition, as single-dose and multiple-dose regimens of the iso-[131I] SGMIB-VHH_1028 are well tolerated, this radiolabeled VHH has a significant ability to prevent tumor progression and increase survival [73].

In another study conducted by Feng et al., the therapeutic use of two sdAbs, VHH_1028 and 5F7, both binding to domain IV of HER2, was assessed. These single-domain antibodies were labeled with iso-211At-SAGMB (N-succinimidyl-3-211At-astato-5-guanidinomethyl benzoate). The cytotoxicity of two radiolabeled antibodies (VHH_2001 and 5F7) was evaluated on the BT474 cell line as HER2-expressing breast cancer cells. VHH_2001 is an HER2-irrelevant nanobody used as a control. To evaluate the effectiveness of these radiolabeled single domain antibodies (iso-211At-SAGMB-VHH_1028, iso-211At-SAGMB-5F7, iso-211At-SAGMB-VHH_2001 and 211At-SAGMB-VHH_1028), their effect on mice with BT474 subcutaneous xenografts was performed. According to the result, exposure of BT474 cells to iso-211At-SAGMB-5F7 decreased their clonogenic survival, but iso-211At-SAGMB-VHH_2001 had no significant effect. Moreover, dose-dependent inhibition of tumor growth was observed when administering 5F7 and VHH_1028 labeled with 211At, while this effect was not observed in the case of VHH_2001. Complete tumor regression at a 3.0-MBq dose was reported in 75% and 73% of mice treated with iso-211At-SAGMB-5F7 and iso-211At-SAGMB-VHH_1028, respectively. The increase in average survival was about 49% and 41%, respectively. To summarize, the combination of HER2-targeted sdAbs and iso-211At-SAGMB shows promise for HER2-expressing cancer treatment [74].

Considering that a nanobody can be used to determine HER2 status in breast cancer patients before trastuzumab treatment, Pruszynski et al. produced a radioiodine-bound HER2-specific nanobody (5F7GGC) and evaluated it for targeting HER2-expressing tumors. In this study, the 5F7GGC nanobody was separately attached to 125I and 131I. The binding of the nanobody to iodine 125 was done directly and to iodine 131 indirectly by [131I] IB-Mal-D-GEEEK. Next, using a paired label internalization assay by means of BT474M1 cells and tissue distribution experiments in athymic mice with BT474M1 xenografts, the efficacy of these two radiopharmaceuticals was compared. Radiochemical yields for 131I-IB-Mal-D-GEEEK-VHH and 125I-VHH were about 60% and 84%, respectively. These radiolabeled proteins retained immunoreactivity to bind to tumor cells in vivo and in vitro. Nanobody radiolabeled with [131I] IB-Mal-D-GEEEK showed faster blood clearance, lower non-target organ accumulation (excluding kidneys), simultaneous high tumor-to-tissue and tumor-to-blood ratios, and higher tumor uptake (4.65 ± 0.61% ID/g at 8 h) compared to directly labeled Nb (VHH-I125). The tumor uptake for the I125 radiolabeled nanobody was 2.92 ± 0.24% ID/g at 8 h. Results showed 5F7GGC anti-HER2 Nb with [131I] IB-Mal-D-GEEEK had better tumor targeting than directly labeled Nb, indicating its potential for SPECT and PET imaging of HER2 + tumors [75].

HER2-targeting VHHs, when labeled with α-particle irradiators, offer an appreciated tool for radio immunotherapy that delivers highly localized and mortal radiation to target cells. Pruszynski and colleagues developed the anti-HER2 VHH that was conjugated with p-SCN-Bn-DOTA and labeled with α- irradiators (225Ac) with a 90% yield and about 95% purity. The “p-SCN-Bn-DOTA” is 2-(4-isothiocyanatobenzyl)-1, 4, 7, 10-tetraazacyclododecane-1, 4, 7, 10-tetraacetic acid. 225Ac-DOTA-VHH had a high binding affinity (4.12 ± 0.47 nM) with an over-80% immunoreactivity fraction. Negligible and significant binding of this nanobody to MDA-MB-231 cells (low HER2 expression) and SKOV-3 cells (HER2 overexpression), respectively, confirmed the binding specificity of the nanobody. Also, non-competitive binding to HER2 was done in the presence of excessive trastuzumab. The toxicity of the anti-HER2 radiolabeled nanobody was assessed in vitro. According to the result, this radiopharmaceutical was toxic in an HER2-mediated and dose-dependent manner. IC50 values were 322.1 and 10.2 kBq/mL for 225Ac-DOTA control and 225Ac-DOTA-VHH, respectively, on SKOV-3 cells. Moreover, the IC50 of 225Ac-DOTA-Nb on MDA-MB-231 cells was 282.2 kBq/mL. Biodistribution studies in ex vivo conditions were done in mice with high and low HER2-expressing tumors. The results showed fast uptake in SKOV-3 tumors (~ 4%ID/g after 2 h) compared to MDA-MB-231 tumors (~ 0.5%ID/g after 2 h), and high tumor-to-normal tissue ratios. The cell-associated fraction of 225Ac-DOTA-VHH was about 35% over 24 h. About a 70% reduction in renal retention was achieved by co-injection of 225Ac-DOTA-VHH with gelofusine. 225Ac-DOTA-Nb is a promising radiolabeled drug for targeted α-particle cancer therapy [76].

A study by Puttemans et al. investigated the therapeutic efficacy of 225Ac DOTA 2Rs15d for brain metastatic breast cancer alone or in combination with trastuzumab. In this study, the 2Rs15d nanobody was radiolabeled with 131I, 111In, and 225Ac. Therapeutic efficiency was determined for the 225Ac and 131I-labeled nanobodies and compared to trastuzumab in an HER2-positive mouse tumor model. In contrast to radiolabeled trastuzumab, which was unable to accumulate in intracranial SKOV3 tumors, radiolabeled nanobody (225Ac DOTA 2Rs15d) demonstrated strong and targeted tumor uptake in HER2-positive brain lesions [77].

In a study by Xavier and colleagues, anti-HER2 nanobody labeling with 18F and its validation for in vivo assessment was carried out. Anti-HER2 VHH was conjugated with the prosthetic group, [18F]-SFB. In the following, specificity, and tumor targeting were assessed in mouse model bearing SKOV3 tumor cell. According to in vivo investigations, HER2 positive xenografts with high tumor to muscle and tumor to blood ratios produced high contrast PET imaging with a high selective uptake. Considering that the 18F-FB-anti-HER2 can image HER2-expressing tumors when prescribed simultaneously with (Herceptin), it is possible to simultaneously use imaging with this nanobody in patients treated with Herceptin. Interestingly, this probe showed rapid renal clearance, and therefore has great potential for imaging HER2-overexpressing tumors [78].

Another form of 5F7 nanobody radiolabeling tag was developed by Choi et al. The nanobody was labeled with two synthetic agents, iso-[211At]-SAGMB or [211At]-SAGMB, and was assessed in the BT474 subcutaneous xenograft mouse model. Although both radio-conjugate nanobodies showed the same purity and in vivo behavior in relation to non-specific accumulation in the lungs and spleen, the stability of these two nanobodies in in vivo conditions was different according to the type of isomer. Higher binding affinity and tumor uptake were demonstrated by iso-[211At]-SAGMB-5F7. It also had shorter renal retention than [211At]-SAGMB-5F7 and a greater tumor-to-background ratio. As a result, the iso-conjugate showed greater promise and was examined in more detail in a different investigation using 211At-labeled nanobodies [79].

In a study by D’Huyvetter et al., a nanobody was labeled with Lutetium-177. To choose the optimal chemical bond between the 2Rs15dHIS nanobody (anti-HER2) and radioisotope, four different bifunctional chelators (DOTA-NHS ester, p-SCN-Bn-DOTA, 1B4M-DTPA, or CHX-A"-DTPA) were compared with each other. All the conjugates studied had great stability over time, but the 2Rs15d bonded to the 1B4M-DTPA was determined to be the best molecule because it had the lowest background uptake and a high specific tumor uptake [80]. In a supplementary study by the same group, the effect of the presence of a C-terminal tag in the structure of the nanobody on the performance of the radio-conjugated nanobody was investigated. In this study, which was investigated on xenografted HER2-positive mice (with SKOV3 cell line), nanobodies with different C-terminal amino acid tag sequences (His-tagged, Myc-His-tagged, and untagged) were used. According to the results, unlabeled 177Lu-DTPA-2Rs15d showed the highest tumor-specific absorption and the lowest background tissue and organ absorption. Also, among the investigated nanobodies, the lowest renal retention was observed in unlabeled 177Lu-DTPA-2Rs15d. Also, in order to compare the nanobody with the complete antibody, trastuzumab antibody was also conjugated by Lutetium-177. When the data were compared, it was found that the tumor received a six-fold larger dose of 177Lu-DTPA-trastuzumab than unlabeled 177Lu-DTPA nanobody. However, there was a notable retention of radioactivity in the bone, liver, lung, blood, and spleen when 177Lu-DTPA-trastuzumab was used. However, histological investigations revealed no signs of nephrotoxicity, and 177Lu-DTPA-2Rs15d treatment led to nearly total tumor growth inhibition [81].

In a study by Vaneycken et al., an anti-HER2 nanobody was developed. The 2Rs15d nanobody was stable, showed low nanomolar affinity, and did not compete with two important anti-HER2 therapeutic antibodies (Trastuzumab and Pertuzumab). In the following, 2Rs15d technetium-99m was established as a radiolabeled VHH for HER2-positive cancer imaging. The results demonstrated that at one hour following intravenous injection, 2Rs15d-99mTc had a high concurrent tumor-to-muscle (49.6 ± 11.8 at 1 h p.i.) and tumor-to-blood (16.4 ± 3.6 at 1 h p.i.) ratio, low accumulation in nontarget organs other than the kidneys, strong tumor uptake in HER2 positive tumor models, and rapid blood clearance [71].

The safety, dosimetry, and effectiveness of 99mTc-labeled anti-HER2 nanobodies in diagnostic imaging of HER2 in patients with breast cancer are presently being assessed in a phase I clinical trial (NCT04406686). The outcomes of HER2 expression as determined by FISH and biopsy tissue immunohistochemistry will then be compared with the molecular imaging results. Since 99mTc is easily available in all nuclear medicine units through a generator system, and on the other hand, its labeling is an easy and quick operation, this radionuclide is very suitable for cancer therapy. Furthermore, the half-life, about 6 h, corresponds with the quick circulation elimination of nanobodies, enabling strong contrast early diagnostic SPECT pictures [82].

In a study by Xavier et al., the anti-HER2 nanobody (2Rs15d) was labeled with 68Galium via NOTA (1, 4, 7-triazacyclononane-1, 4, 7-triacetic acid) and evaluated for its usage for HER2 iPET imaging. Biodistribution studies showed fast and specific uptake in HER2-positive tumors (4.34 ± 0.90% IA/g) and high tumor-to-muscle and tumor-to-blood ratios at 1 h after injection. 68Ga-NOTA-2Rs15d, a novel anti-HER2 PET tracer, was created quickly and in mild conditions. High-specific-contrast imaging of HER2-positive tumors was demonstrated in preclinical validation, and no toxicity was noted [72]. Keyaerts and colleagues conducted the clinical study of the 68Ga-NOTA-2Rs15d (EudraCT 012001135-31). The results showed that PET/CT by radiolabeled nanobody (68Ga-HER2-Nanobody) is a safe method with a radiation dose comparable to other PET detectors and can be used routinely. The biodistribution of 68Ga-NOTA-2Rs15d is promising, with the highest uptake in the intestines, liver, and kidneys. However, the results showed very low background levels in other organs that are usually the site of primary breast carcinoma or tumor metastasis. When compared to normal surrounding tissues, the amount of tracer accumulation in the metastases of HER2-overexpressing patients is significant, which calls for additional evaluation in a phase II trial. A second phase II clinical trial (VUBAR) is examining the relationship between image-based HER2 assessment following uptake of 68Ga-NOTA-2Rs15d in patients with local or distant breast cancer metastases and the outcomes of a biopsy of the same lesion [83].

In the study by Zhao, a 131I-labeled anti-HER2 nanobody was generated. 131I-NM-02 was prepared with satisfactory radiochemical purity and stability in laboratory conditions. In HER2-positive tumor-bearing mice, there was evident tumor absorption along with quick blood clearance and advantageous biodistribution. With good organ compatibility, 131I-NM-02 might prolong the lives of these mice and greatly limit the formation of tumors. 131I-NM-02's inhibitory effects and negligible tumor accumulation were noted in the negative control group. It may be possible to investigate 131I-NM-02 as a novel technique for targeted radionuclide therapy of HER2-positive breast cancer [84].

In a phase I clinical trial study (NCT02683083), 131I-GMIB-Anti-HER2-VHH1 was assessed in breast cancer patients. The radiolabeled VHH consists of an anti-HER2 nanobody connected via the SGMIB to therapeutic 131I. The objective of the phase I trial was to assess the biodistribution, tumor imaging potential, safety, and radiation dosimetry of the 131I-GMIB nanobody in both healthy volunteers and breast cancer patients. The biologic half-life of the radiolabeled nanobody was about 8 h in healthy subjects. 131I-GMIB-VHH was eliminated from the blood with a 2.5-h half-life after intravenous administration. There was no increased accumulation of the compound in the thyroid or stomach, and its level in the blood was stable. SPECT/CT imaging in patients with advanced breast cancer showed focal uptake of 131I-GMIB-anti-HER2-VHH1 in metastatic lesions. Given its distinct mode of action from pertuzumab, trastuzumab, or trastuzmab emtansine, this radiopharmaceutical can provide patients who have progressed on these treatments with new therapeutic choices due to its good toxicity profile and uptake in HER2-positive lesions. A follow-up phase I/II research project (NCT04467515) is scheduled to evaluate the therapeutic window of this drug through a dose escalation [85] (Table 3).

**Table 3:** Studies conducted in the field of targeted drug delivery by radiolabeled nanobodies

Name of NDs	Antigen	Type of radiolabeling	Type of study	The mechanism of action	References
Iso-[131I]SGMIB-VHH-1028	HER2	Iso-[131I] SGMIB	Pre-clinical/ Balb/c mice bearing BT474 tumor cells	Prevent tumor progression	Feng et al. [73, 74]
Iso-211At-SAGMB-5F7	HER2	Iso-211At-SAGMB	Pre-clinical/ Balb/c mice bearing BT474 tumor cells	Tumor regression	Feng et al. [73, 74]
125I-VHH 131I- IB-Mal-D-GEEEK-VHH	HER2 HER2	Iodine125 IB-Mal-D-GEEEK/Iodine 131	Pre-clinical/ Balb/c mice bearing BT474 tumor cells	Using in radio-immunotherapy and SPECT and PET imaging of HER2 + tumors	Pruszynski et al. [75, 76]
225Ac-DOTA- 2Rs15d VHH	HER2	p-SCN-Bn-DOTA/α- irradiators 225Ac	Pre-clinical/ Balb/c mice bearing SKOV3 tumor cells	Using in radio-immunotherapy in HER2 + tumors	Pruszynski et al. [75, 76]
225Ac DOTA 2Rs15d	HER2	225Ac	Pre-clinical/ Balb/c mice bearing SKOV3 tumor cells	Strong and targeted tumor uptake in both HER2 positive brain lesions	Puttemans et al. [77]
18F-FB-anti-HER2	HER2	4-[18F] fluorobenzoate ([18F]-SFB)	Pre-clinical/ Balb/c mice bearing SKOV3 tumor cells	A radiometal labeled nbs when imaging lesions in the proximity of the kidneys	Xavier et al. [72, 78]
Iso-211At-SAGMB-5F7	HER2	Iso-[211At]SAGMB	Pre-clinical/ Balb/c mice bearing BT474 tumor cells	Targeted radiotherapeutics for the treatment of HER2 expressing malignancies	Choi et al. [79]
177Lu-1B4M–DTPA-2Rs15dHIS	HER2	177Lu-1B4M–DTPA	Pre-clinical/ Balb/c mice bearing SKOV3 tumor cells	Radiolanthanide Lutetium-177 emit radiation with an appropriate linear energy transfer for the destruction of tumor cells	D’Huyvetter et al. [80, 81, 85]
99mTc-2Rs15d	HER2	Technetium-99m	Phase I clinical trial (NCT04040686)	HER2-positive tumor imaging	Devoogdt et al. [82] Vaneycken et al. [69, 71]
68Ga-NOTA-2Rs15d	HER2	Gallium 68	Phase I clinical trial (EudraCT 012001135-31) A second phase II clinical trial (VUBAR)	A safe HER2-positive tumor imaging used as PET radiotracers	Keyaerts et al. [83] Xavier et al. [72, 78]
131I-NM-02	HER2	Iodine 131	Pre-clinical/ Balb/c mice bearing SKBR3 tumor cells	A novel technique for targeted radionuclide therapy of HER2-positive breast cancer	Zhao et al. [42, 84]
131I-SGMIB-2Rs15d	HER2	Iodine 131	Phase I clinical trial (NCT02683083) A follow-up phase I/II clinical trial (NCT04467515)	Therapeutic potential for patients who progress on trastuzumab, pertuzumab, or trastuzumab emtansine	D’Huyvetter et al. [80, 81, 85]

### Nanobody based immunotoxin

**Fig. 3:**
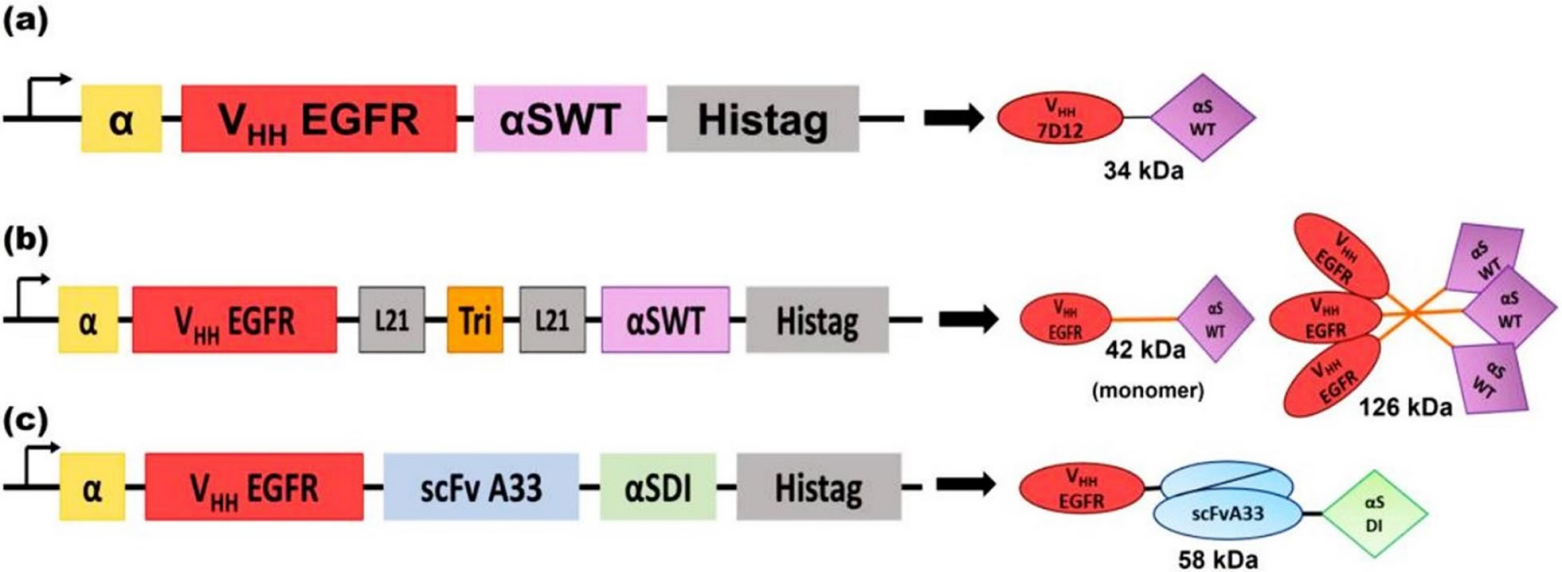
An arrangement diagram of three types of nanobodies and immunotoxin: nanoITXs VHHEGFRαS (**a**), TriVHHEGFRαS (**b**), and BsITX [89]

Nanobody constructs conjugated with immunotoxins as protein-based anticancer agents provide both cytotoxic and molecular-targeted cancer therapy. The successful application of nanobody-based immunotoxins has inspired the improvement of therapeutic novel nobodies and their derivations. Immunotoxins, as chimeric molecules, have played key roles in various malignancy treatments [86]. During the last few years, to achieve efficient cancer-targeted imaging and a controlled delivery vehicle, nanobody targeting cadherin 17 showed theranostic potential in gastric cancer. The assessment of the E8 nanobody against domains 1–3 of cadherin 17 coupling with pseudomonas exotoxin 38 showed outstanding inhibition within in vitro and in vivo studies. This ability can be overcome by treatment-resistant cancer cells and other main drawbacks of chemotherapy [87]. Given that more attention is paid to epidermal growth factor receptors (EGFR) in many epithelial cancers, EGFR nanobodies are used in EGFR-overexpression cancer cell treatment. Recombinant immunotoxins and EGFR nanobodies pave the way to increase treatment efficiency and dominate cell drug resistance obstacles. The humanized biparatopic nanobody PE24 against EGFR-positive cancers could be applied as a promising candidate to target EGFR-positive tumors [88]. In the next generation of nanobody-based immunotoxin design, researchers introduced three recombinant proteins with the target domain against EGFR (VHH 7D12) and immunotoxin moieties. The different investigations on linkage nanobody and immunotoxin, including VHH 7D12 and the fungal ribotoxin α-sarcin (nanoITXs VHHEGFRαS), trimerization domain (TIEXVIII) into the construct (TriVHHEGFRαS), and bispecific immunotoxin (BsITX) (Fig. 3) established that α-sarcin-based nanoimmunotoxin have high-quality therapeutic effect. This study supports their massive and strong capacity for the diagnosis and treatment of colorectal cancer [89].

## Conclusion

In comparison with other targeted drug approaches, including whole antibodies, scFv, and Fab, nanobody molecules are the priority queue for art-of-state target therapy. The advantages of nanobody structures have led scientists to further study and focus on the design of nanobody products. The essence of attention to nanobodies is their small size due to their single domain, greater physicochemical stability, better tissue penetration, rapid clearance from blood, and being produced in bacterial or yeast-expression hosts. Unlike nanobodies, which are constructed only from heavy chains, scFv and Fab are established from the VL and VH domains. 12–15 kDa size of nanobody, leading to manipulation easily against ~ 25 kDa size of scFv, ~ 50 kDa size of Fab, and 150 kDa size of whole antibody. Due to their minimal size, nanobodies have the ability to be conjugated to molecules and penetrate tissues for greater effect in treatment. Also, the development of nanobodies is more affordable, and there is no need for a mammalian expression host. Notwithstanding these exciting prospects, nanobody-based therapy faces unfavorable obstacles. One of these includes low specificity and off-target effects on some targets, leading to inappropriate binding to conserved sites through various enzymes [7, 90]. To overcome the previous obstacles, nanobodies with bispecific characterization can improve the target of tumors and, moreover, provide both tumor antigen and T lymphocytes as a promising antitumor therapy [91]. Another challenge of using nanobodies is their short half-life in the blood, which is also due to their small size and renal filtration and clearance. However, because of their quick renal clearance, they have a short blood half-life, which typically means frequent dosage and poor therapeutic efficacy [92]. Considering that the molecular weight of nanobodies is less than the size of the glomerular filtration cutoff (65 kDa), the use of these molecules in certain clinical treatments that require antibody circulation for long periods of time will cause problems. Although this small size can be a beneficial feature due to its high tissue permeability [93]. Bispecific VHHs can contribute to their stability by overcoming the short serum half-life of VHHs. So far, several techniques have been developed to increase the half-life of VHHs, including the use of stabilizing groups such as polyethylene glycol (PEG) molecules and fusion with serum proteins such as albumin [93]. Another important challenge related to nanobodies is the use of biohazardous substances such as bacteriophages, plasmids, antibiotics, recombinant DNA, etc. in their production process. As a result, extreme caution should be used when handling these materials, and strict procedures for disposing of waste should be followed (such as autoclaving solid waste, adding bleach to liquid waste, decontaminating using disinfectants, and handling materials by personnel who have received the necessary training and protective gear) [92].

Despite many desirable properties of nanobodies, the non-human origin of nanobodies may cause unwanted immune responses and ADA (anti-drug antibody) production if they are prescribed as therapeutic agents; however, this problem is much more challenging when using whole antibodies. One of the ways that we can solve this concern is by genetically engineering camel VHHs so that FRs (frameworks) of camel origin are replaced with similar human parts. But it seems that this method is not so practical. The reason for this inefficiency is that although switching from fully murine to chimeric (constant domain from human and VH/VL domain from mouse) antibody treatment meaningfully inhibited anti-drug antibody (ADA) production, more manipulations such as humanized antibodies and fully human antibodies have had a relatively minor effect on immunogenicity. At high intravenous doses, even fully human antibodies can cause ADA. Although the engineering of camel nanobodies based on the variable parts of human antibodies can be exciting considering the structural differences of these antibodies [94]. Another disadvantage of nanobodies is the lack of Fc section. However, without Fc, nanobodies are unable to activate complement and the ADCC pathways, which means they are ineffective against cancer cells. Therefore, tying them to therapeutic molecules and other drug carriers can expand their application in the treatment of cancer. Due to their simple ratio and small dimensions, molecules that bind to nanobodies don’t significantly alter their structural integrity.

In general, nanobodies have the ability to target drug delivery with three methods. These three methods were: direct binding of drugs to nanobodies; binding of nanobodies to carriers containing drugs; and binding of nanobodies to radioactive substances. The studied studies show that due to the small size of nanobodies, their direct connection to large molecules such as protein toxins may cause the folding of nanobodies to be disrupted, and their specific binding will be a challenge. But connecting to carriers containing drugs, because it happens through a linker, will have less effect on the folding of nanobodies. Also, considering that the half-life of nanobodies in the body is short, the use of radiolabeled nanobodies has made more progress, and studies in this field have entered the clinical phases. It seems that in the not-so-distant future, more of these radiopharmaceuticals will be available in the clinical phases.

## Data Availability

Not applicable.

## Abbreviations

HER2: Human epidermal growth factor receptor positive
ER: Estrogen receptor positive
PR: Progesterone receptor positive
mAb: Monoclonal antibodies
NK cells: Natural killer cells
Nbs: Nanobodies
ADC: Antibody drug conjugate
NDC: Nanobody drug conjugate
FR: Frame work
CDR: Complementary determining region
EGFR: Epidermal growth factor receptor
ScFv: Single chain fragment variable
PEG: Polyethylene glycol
LF: Lethal factor
LT: Lethal toxin
MAPK: Mitogen activated protein kinase
PDT: Photodynamic therapy
PS: Photosensitizer
SGMIB: N-succinimidyl 3-guanidinomethyl 5-[131I] iodobenzoate
TNF: Tumor necrosis factor
MUC1: Transmembrane glycoprotein Mucin 1
BLBC: Basal like breast cancer
BM: Brain metastasis
DR: Death receptor
LGHMGA: Actic acid-co-glycolic acid-co-hydroxymethyl glycolic acid
PCI: Photochemical internalization
DSPE: Di-stearoyl phosphatidyl ethanolamine
NIT: Nitroxoline
CIS: Cisplatin
Mal: Maleimide
QD: Quantum dot
AF: Aminoflavone
TNBC: Triple negative breast cancer
FITC: Fluorescein isothiocyanate
PEI: Polyethyleneimine
